# Prevention of cisplatin-induced hearing loss in children: achievements and challenges for evidence-based implementation of sodium Thiosulfate

**DOI:** 10.3389/fonc.2024.1336714

**Published:** 2024-03-18

**Authors:** Annelot J. M. Meijer, Franciscus A. Diepstraten, Marry M. van den Heuvel-Eibrink, Archie Bleyer

**Affiliations:** ^1^ Princess Máxima Center for Pediatric Oncology, Utrecht, Netherlands; ^2^ Wilhelmina Childrens Hospital, University of Utrecht, Utrecht, Netherlands; ^3^ Radiation Oncology, Knight Cancer Institute, Oregon Health and Science University, Portland, OR, United States

**Keywords:** ototoxicity, sodium thiosulfate, pediatric cancer, prevention, hearing loss, cisplatin

## Abstract

Ototoxicity is a devastating direct, irreversible side effect of platinum use in children with cancer, with its consequent effect on speech, language and social development, quality of life and adult productivity. Cisplatin, an essential chemotherapeutic agent for the treatment of solid tumors in children, is a DNA cross-linking agent. Which causes hearing loss in 50-70% of cisplatin treated children. Fortunately, to prevent hearing loss, sodium thiosulfate (STS), which binds to cisplatin, and reduces the superoxides in both tumor and outer hair cells of the cochlea has now been discovered to be an effective and safe otoprotectant if administered correctly. The aim of this perspective paper is to explore the key safety issues and challenges important for pediatric oncologists and pharmacists when considering the clinical use of STS as an otoprotectant for children and adolescents receiving cisplatin. These include: the choice of the formulation; the timing, both that of the STS in relation to cisplatin as well as the timing of the cisplatin infusion itself; the dosing; the challenge left by the definition of localized versus disseminated disease and the difference in indication for STS, between cisplatin treated patients and those receiving another platinum chemotherapeutic agent, carboplatin.

## The choice of the formulation

There is only one formulation authorized for clinical use as an otoprotectant in children. This specific STS formulation, designed for use in children, has received marketing authorization for the prevention of permanent, life-impacting, cisplatin-induced hearing loss (CIHL) with the trademark name of Pedmark™ in the USA and Pedmarqsi™, in Europe, (this product will be referred to as PM hereafter). PM is approved for cisplatin treated children aged 1 month to 18 years with localized solid tumors by the Food and Drug Administration (FDA), European Medicines Agency (EMA), and Medicines and Health Research Authority (MHRA), in 2022 and 2023, respectively. PM’s unique formulation of STS has been found to be safe and effective based on evidence from two international randomized phase 3 pediatric clinical trials, including patients with localized disease (1, 2). Apart from PM, other readily available STS compounds are being considered for clinical use for ototoxicity prevention. Clinical trials, with these latter compounds have not been pursued, and these products have therefore not been approved by the FDA, EMA or MHRA for otoprotection. The approved PM formulation does not contain potassium chloride and has low levels of boron. Alternative products, that have not been clinically tested for safety and efficacy in children, are likely to contain potassium chloride and/or higher levels of boron (3). Administering such formulations at the dose and rate of administration required for CIHL prevention is not desirable without any further testing, for safety reasons. One particular STS (also potassium and boron containing) product has been licensed for children, but only for the indication of cyanide poisoning, and at a much lower dose and only for single, non-repetitive administration purposes (4). Cyanide poisoning is fortunately rare, especially in children, whereas cisplatin is repeatedly administered in most oncology protocols; sometimes even up to 30 times (5). Therefore, we cannot extrapolate the safety of this particular formulation of STS specifically approved for cyanide poisoning to its use for otoprotection.

## The timing of STS administration relative to the cisplatin infusion is critical

STS has previously been used in adult studies to prevent cisplatin-related renal toxicity and for this indication, STS was administered at the same time as cisplatin (6, 7). It is important to differentiate these early studies from subsequent studies in children aimed at reducing CIHL. Administration of a high dose of STS too early (i.e. at the beginning of or during the cisplatin infusion), can potentially reduce the efficacy of the cisplatin anti-tumor treatment (8). However, in preclinical studies, there have been conflicting reports regarding the impact of STS on the efficacy of cisplatin (9, 10). More recent studies have provided evidence suggesting that delayed administration of STS does not compromise the antitumor activity of cisplatin (11, 12). Administering STS too late after the cisplatin infusion, however, reduces the otoprotection it can provide (13). The prescribed 6-hour delay after the end of the cisplatin infusion provides both a safe and effective time window as confirmed by preclinical studies (12, 14) and it was this strategy which was used in the randomized international pediatric clinical trials (**Table 1**) (1, 2). (It is important to realize that this 6-hour delay is too late to reduce cisplatin-induced renal toxicity, since cisplatin will have already have been grossly eliminated by the kidney. However, renal function protection can be achieved by forced diuresis using mannitol before, during and following the cisplatin infusion (15).

**Table 1:** Results of pursued RCTs in pediatric solid tumors.

Author, year	Study	Pediatric Patients n=, strata	STS infusion time (minutes)	Time CP-STS (hrs)	Results hearing loss	Survival	
						3 yrs EFS	3yrs OS
Freyer D, 2017*	ACCL0431	49 CP+STS 55 CP Mixed Solid Tumors	15	1-6	14/49 (28,6%) 31/55 (56,4%) (P 0.0036)	54% 64%	70% 87%
Brock P, 2018**	SIOPEL6	57 CP+STS 52 CP Standard-Risk Hepatoblastoma	15	6	18/55 (33%) 29/46 (63%) (P 0.002)	82% 79%	98% 92%

EFS, Event free survival; OS, Overall survival; hrs, hours; yrs, years; CP, Cisplatin; STS, Sodium thiosulphate (the STS formula in these two trials was that produced by Fennec Pharma now licensed as PedmarkTM in the US and PedmarqsiTM in Europe, further referred to as PM). *Randomization stratification: Age and length of cisplatin infusion, all children treated according to local physician decision. **Randomisation stratification: Pre Treatment Extent of disease, age and country, all children treated uniformly on trial.

To optimally preserve hearing, it is advised to administer STS after each cisplatin administration, particularly the first dose, since permanent cochlear damage can already occur at the first cycle (16). STS is advised to be administered intravenously over 15 minutes, 6 hours after the end of the cisplatin infusion.

## The length of the cisplatin infusion

Cisplatin kills both tumor cells as well as sensitive normal cells, (particularly the outer hair cells) of the inner ear or cochlea, by creating radical superoxides inside the cell (17, 18). It is known to accumulate in the cochlea for months to years (19). International efforts are now being undertaken to implement STS as otoprotectant in clinical pediatric oncology practice, and in that context infusion duration of cisplatin is an important issue. In contrast to the Children’s Oncology Group (COG) cisplatin containing treatment protocols, where 1-6 hour infusions are standard of care in solid tumor protocols, a limited number of protocols worldwide still include long lasting cisplatin infusions occasionally even up to 96 hours (20). Cisplatin was introduced into pediatric patient care in the 1980’s and was found to be the most emetogenic treatment of all chemotherapeutic agents (21). It was rapidly discovered that slowing the infusion time helped to alleviate both nausea and vomiting. Historically, this tendency became written into treatment protocols in the years before serotonin antagonists became available as anti-emetics (22), and for many tumor types has not been subsequently addressed. However in the International Society of Pediatric Oncology Epithelial Liver Tumor Group (SIOPEL) randomized standard risk trial, cisplatin became the key chemotherapeutic agent for the treatment of hepatoblastoma (23). When SIOPEL launched its randomized follow on clinical trial, SIOPEL6, the cisplatin infusion time was reduced from 48 hours (SIOPEL1-5) to 6 hours, and this proved to be safe, i.e. the 3 year overall survival in patients receiving cisplatin, followed 6 hours later by a 15-minute infusion of STS in SIOPEL6, was 98%, which was comparable to the overall survival in the previous SIOPEL3 trial in equivalent-risk patients. This change in infusion timing also did not increase the renal toxicity that occurred in SIOPEL3 (23). When the subsequent Pediatric Hepatic International Treatment Trial (PHITT) was designed, the excellent survival evidence from COG American hepatic tumor studies was used to internationally agree the 6hr infusion time of cisplatin for all patients. Hence, to implement STS otoprotection for all children with cancer, treatment protocols globally will need to be adapted, with particular regard to cisplatin infusion time. These necessary protocol amendments need to be considered and applied by agreement and consensus from the various international tumor group protocol committees.

## Dosing

Although Pedmark™ (FDA) and Pedmarqsi™ (EMA and MHRA) are identical products and formulations the licensing authorities have applied different approaches to the dosing calculations of this aqueous solution of STS. To clarify this, Pedmark™ has been licensed as a ‘pentahydrate equivalent’ molecule and uses the molecular weight of the pentahydrate salt (125 mg/ml) for the dose calculation, whereas Pedmarqsi™ has been licensed as the anhydrous salt and uses the molecular weight of the anhydrous salt for the dose calculation. With Pedmark™, children over a body weight of 10 kg are prescribed 20 g/m^2^, between 5 and 10 kg, 15 g/m^2^ and in children with a body weight less than 5 kg, 10 g/m^2^ (**Table 2**). The dosages found in the prescribing information are concordant with the dosages used and published in SIOPEL6 (1). At the time of the two clinical trials, the yet to be approved STS formulation was manufactured in pentahydrate powder form which needed to be diluted 1:1 in sterile water to make the aqueous solution (1, 2). The current manufacturing process uses the same formula but provides an already prepared aqueous solution of anhydrous STS. Pedmarqsi™ has been licensed using the molecular weight of the anhydrous salt as an 80 mg/ml anhydrous solution. The dose for a child with a body weight over 10 kg is 12.8 g/m^2^, between 5 and 10 kg, 9.6 g/m^2^ and less than 5 Kg, 6.4 g/m^2^ (**Table 2**). Although the FDA and EMA/MHRA have chosen to use different dosing calculations, the ototoxicity preventative dose received by the child is identical.

**Table 2:** Formulation and dosing of Pedmark™ and Pedmarqsi™.

	Pedmark™	Pedmarqsi™
	FDA approved	EMA and MHRA approved
	pentahydrate salt	anhydrous salt
Dosing based on body weight		
< 5 kg	10 g/m^2^	6.4 g/m^2^
5-10 kg	15 g/m^2^	9.6 g/m^2^
> 10 kg	20 g/m^2^	12.8 g/m^2^

EMA, European Medicines Agency; FDA, Food and Drug Administration; MHRA, Medicines & Healthcare products Regulatory Agency.

## Localized versus disseminated disease

There is debate regarding the use of STS for otoprotection in both local and disseminated diseases, the following paragraph outlines the rationale for this. To date, while both randomized clinical trials included children with localized disease, only the COG randomized trial included children with disseminated disease (2), and so marketing authorization for PM was requested and approved only for use in children with localized tumors. The original analysis of the ACCL0431 trial which was stratified only by age and length of cisplatin infusion time showed no significant difference in survival outcomes between the observation and STS randomized arms. However, the COG carried out a *post hoc* analysis by asking the local physicians several questions, the answers to which were not centrally reviewed. One question asked whether patients had localized or disseminated disease. The survival was then reanalyzed according to the local physician’s report. There were 77 patients with localized disease and no statistically significant difference in Event Free Survival or Overall Survival was found in this group. However in the 47 patients reported to have disseminated disease, the overall survival rate in the STS arm was statistically significantly different to the observation arm (2). Recently, the same authors pointed out that the children with disseminated disease who did not receive STS had a better than originally predicted survival (as compared to the literature) and that the difference between the two arms could be due to an imbalance in prognostic groups (24). Even though a biologically plausible rationale for a difference between localized disease and disseminated disease is lacking, only the licensing for localized disease was requested (25). Although the efficacy and safety of STS in disseminated disease requires further study, it is in these children that hearing loss is the most severe (26–29). It is therefore important that a risk benefit analysis be carried out for each child receiving cisplatin to assess the safest approach for any individual child particularly where other sensory organs or brain function may be severely impacted.

## STS in cisplatin versus carboplatin treated children

It is currently not appropriate to administer intravenous STS after intravenous carboplatin, as this has a different pharmacokinetic profile. STS has only been used successfully as an otoprotectant after intra-arterial carboplatin, where the STS is separated by space using a two-compartment blood-brain barrier disruption (BBBD) model, in patients with brain tumors (30). No safety and efficacy data based on clinical trials is available that provides evidence for the use of STS otoprotection in carboplatin exposed children where BBBD is not being applied. With regard to carboplatin, it must be emphasized that the ototoxicity occurs less often, except in patients with accelerated total cumulative dosages, such as for high-dose myeloablative therapy.

## Side effects of STS

The high sodium load of STS is emetogenic (31), therefore anti-emetics, which are standard of care for children receiving cisplatin, are recommended to be given additionally at least 30 minutes prior to STS administration. The nausea usually ends when the 15 minutes STS infusion is completed. Timing of the STS infusion while the child is asleep can be of help, providing that the patient is adequately monitored. The high sodium load does produce a short rise in serum sodium levels and blood pressure, but these return to normal values within 24 hours provided the child is well hydrated. Late toxicity has not been reported. It is important to clarify that, as far as we know, there are currently no published Patient-Reported Outcome Measures (PROMs), Quality of Life (QoL) measures, or qualitative data from patients or their family members who have experienced concurrent STS/cisplatin emetogenesis.

## Conclusion

In conclusion given the 50-70% prevalence rate of CIHL in exposed children (26, 32, 33) and its negative impact on speech and language development, subsequent emotional and social development with consequent impairment of daily functioning and quality of life (34), the reduction of this irreversible serious hearing loss by using STS as the most effective and safe way for cisplatin treatment in children with localized cancers is warranted. Further studies are necessary to confirm the safety of STS in children with disseminated cancer.

## Data availability statement

The original contributions presented in the study are included in the article/supplementary material, further inquiries can be directed to the corresponding authors.

## Author contributions

AM: Conceptualization, Writing – original draft, Writing – review & editing. FD: Project administration, Writing – review & editing. MH-E: Conceptualization, Supervision, Writing – original draft, Writing – review & editing. AB: Writing – review & editing.
